# Evaluating the Content and Quality of Videos Related to Hypertrophic Scarring on TikTok in China: Cross-Sectional Study

**DOI:** 10.2196/64792

**Published:** 2025-04-29

**Authors:** Jiangkun Wang, Kai Xu, Juanjuan Wu, Wen Liang, Weiming Qiu, Song Wang

**Affiliations:** 1 Department of Burns and Plastic Surgery, General Hospital of Central Theater Command Wuhan China; 2 School of Medicine, Wuhan University of Science and Technology Wuhan China

**Keywords:** hypertrophic scars, health education, TikTok, social media, information quality

## Abstract

**Background:**

Hypertrophic scars (HTSs) are a predominant condition after burns and trauma, and it causes severe physiological and psychological problems. TikTok (Douyin in Chinese), a popular platform for sharing short videos, has shown the potential to spread health information, including information related to HTSs. Educating the public to obtain correct information is important to reduce the incidence of physiological and psychological problems caused by HTSs. However, the quality and reliability of HTS-related video content on TikTok in mainland China have not been thoroughly studied.

**Objective:**

This study aims to evaluate the content and quality of short videos related to HTSs on the Chinese version of TikTok (Douyin) and explore the factors related to their quality, providing valuable insights for health information dissemination.

**Methods:**

We collected a sample of 153 TikTok videos in Chinese related to HTSs and categorized them according to video source and content. We evaluated the video content using a coding schema, and a hexagonal radar schema was used to intuitively display the spotlight and weight of each aspect of the videos. We evaluated quality using 4 standardized tools: the modified DISCERN (mDISCERN) questionnaire, the *Journal of the American Medical Association*, the Global Quality Scale (GQS), and the Health on the Net Foundation Code of Conduct. We also explored the potential relationship between video quality and characteristics.

**Results:**

The analysis showed that health care professionals uploaded all videos about treating HTSs, which matched the hexagonal radar model analysis findings. The quality assessment scores for the *Journal of the American Medical Association*, GQS, mDISCERN, and the Health on the Net Foundation Code of Conduct had median values of 1 (IQR 1-2), 2 (IQR 2-3), 2 (IQR 2-3), and 3 (IQR 3-4), respectively, indicating a need to improve the quality and reliability of videos on HTSs. In addition, high-quality videos were more popular, based on metrics such as likes, comments, favorites, and shares (*P*<.001). Interestingly, the time when the videos were uploaded positively correlated with GQS and mDISCERN scores (*r*=0.393; *P*<.001 and *r*=0.273; *P*<.001), while the video length did not significantly correlate with evaluation scores (*P*=.78, *P*=.20, *P*=.07, and *P*=.04).

**Conclusions:**

The quality of TikTok videos related to HTSs is generally moderate. Users should exercise caution when seeking information on HTSs from TikTok. It is advisable to choose videos uploaded by health care professionals from the burn department and the burn plastic surgery department, and in the Chinese context, those produced in first-tier cities and emerging first-tier cities.

## Introduction

### Background

Hypertrophic scars (HTSs) are a common fibrotic skin condition that can develop from various sources, including acute or chronic wounds, deep burns, and surgical incisions [1]. The overall incidence of HTSs ranges from 4% to 16%, but among patients with burns, the prevalence can be as high as 70% [1,2]. A study of Chinese college students reported an incidence rate of HTSs at 5.2% [3]. In high-income countries, around 100 million individuals are affected by HTSs [4]. HTSs can negatively impact a person’s appearance and lead to impaired skin function, joint deformities, and decreased mobility, significantly affecting mental and physical well-being [5]. The annual global cost for HTS care is estimated at nearly US $20.8 billion, with the United States spending about US $4 billion on treatment yearly [6]. The market for HTS and keloid scar treatments is projected to grow, potentially reaching US $37.9 billion by 2026, with a compound annual growth rate of 9.9% [7]. Making lifestyle changes, such as minimizing intense physical labor, avoiding spicy foods, reducing alcohol consumption, and limiting time spent in hot baths, may help lower the risk of developing HTSs [2]. Early detection, diagnosis, and effective treatment are essential for improving patient outcomes and addressing the physiological and psychological issues related to HTSs. Therefore, educating the public about accurate and reliable health information is crucial in reducing the incidence of problems associated with HTSs.

### Health Information in the Digital Era

The rapid advancement of internet technology has transformed how we share and communicate health information [8,9]. Remarkably, around 80% of individuals worldwide rely on online resources to inform themselves about health matters [10,11]. This shift enhances health communication and unprecedentedly empowers patients in their education and decision-making [12]. With easy access to information online, patients are no longer passive recipients; they have become proactive seekers, fully engaged in their health outcomes [13]. In recent years, health education videos designed to inform viewers, individually or collectively, have surged in popularity [14]. Unlike traditional text, videos on social media platforms present information more digestibly and effectively motivate users toward healthier behaviors through compelling visuals [15,16]. Short video platforms have the potential to spread health education widely, but patients may encounter challenges in using these technologies. Patients’ primary concern when searching for online health information is the quality of the information [17]. The rise of numerous content creators and the lack of regulation on these platforms often lead to concerns about the trustworthiness of the medical information shared [18]. When searching for online health information, the quality of the information is a primary concern for patients [17]. For many nonprofessionals, evaluating the quality of online health information sources, especially for patients with lower health literacy levels, is not easy [19]. Due to the varying quality of short video content, patients often struggle to distinguish between true and false information. This can lead to the spread of misleading information, potentially impacting patients’ understanding of their own medical conditions [20]. It is important to recognize that the content creators on video platforms could be (1) patients themselves or (2) health professionals. Experience sharing by patients who have experienced similar conditions can be a handy educational mechanism for other patients or their caregivers [21]. Health professionals, experts in their fields, can also offer helpful advice. However, more must be done to verify the authenticity of the sources (patients or health professionals) producing these videos. This is one of the primary reasons why a lot of misinformation or unverified health information is propagated on social media and video platforms. Therefore, establishing robust oversight of online health videos is crucial to ensure that patients receive reliable and accurate health information.

### This Study

TikTok (ByteDance), or Douyin, its Chinese name, is the leading video social media app in China, captivating a vast audience. Focusing on diverse content, such as food, travel, and education, it has attracted over 750 million daily active users, making it a vital platform for engagement and discovery [22]. During the COVID-19 pandemic, TikTok videos about the SARS‑CoV‑2 garnered 93.1 billion views by July 2020 [17,19]. In addition, videos tagged with #cancer have amassed over 1.1 billion views worldwide [23]. As a platform for disseminating health information, social media has significant differences in video quality and information accuracy. Previous studies have shown that videos published by health care professionals typically have higher scientific validity and credibility [24]. However, the popularity of these videos is often limited by the social influence of the publishers. Videos posted by social media influencers with many followers may attract more viewers and interactions, even if they lack professionalism. In addition, Ming et al [25] found that erroneous information is commonly present in health education videos released by for-profit organizations. This further highlights the necessity of evaluating video quality and authenticity. Previous research has examined the quality of videos on various themes on TikTok, revealing differences in video quality. For instance, videos about *Helicobacter pylori* infection [11], breast cancer [26], liver cancer [20], and inflammatory bowel disease [27] are generally considered unsatisfactory in quality. In contrast, videos related to plastic surgery are deemed satisfactory in quality and reliability [28]. We found many videos about HTSs on Douyin, the Chinese version of TikTok, but the quality of the information presented is yet to be evaluated. To address this research gap, we assessed the content, quality, and reliability of HTS-related videos on TikTok. We examined the relationship between the quality of video content and audience engagement, focusing specifically on interactive indicators, such as likes, comments, favorites, and shares.

## Methods

### Search Strategy and Data Extraction

**Figure 1:**
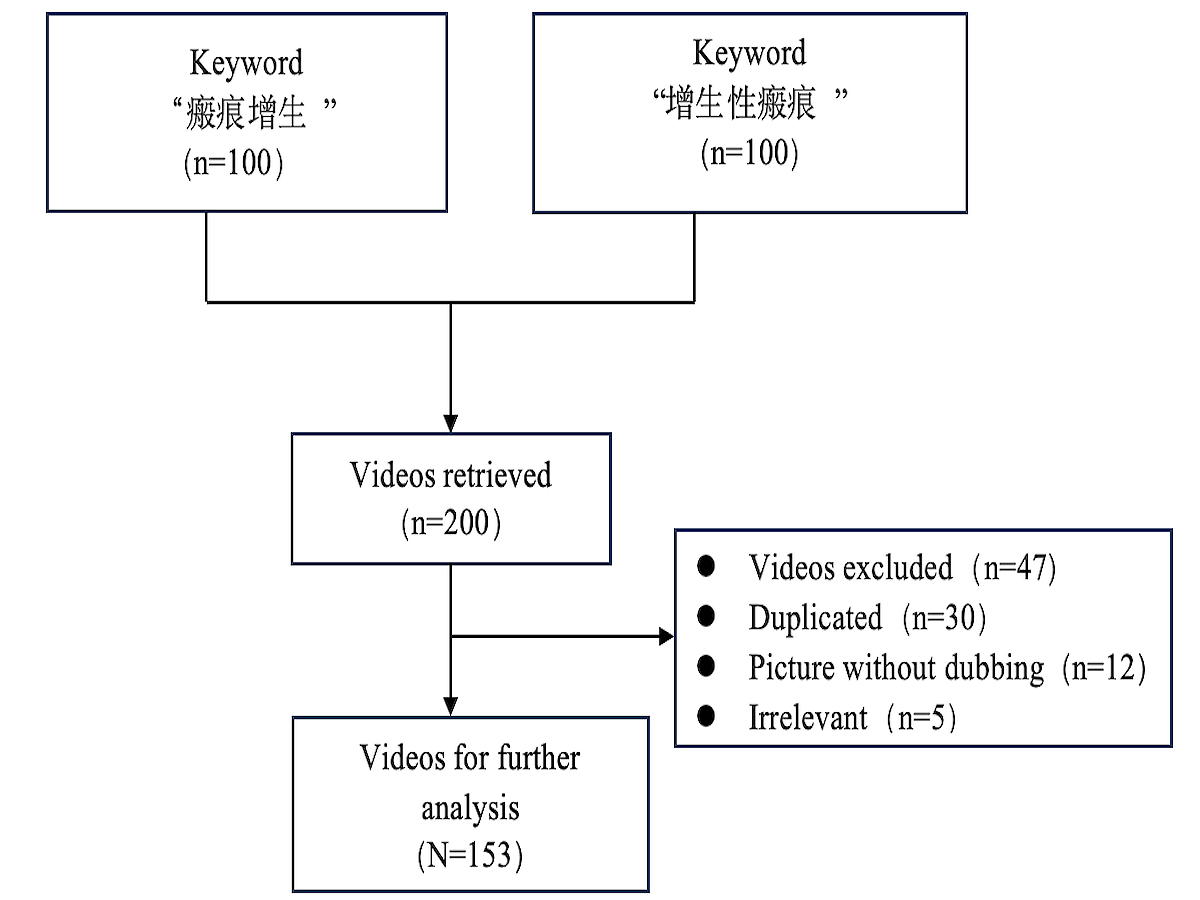
Search strategy for short videos on hypertrophic scars.

In this cross-sectional study, we used the keywords “瘢痕增生” (“scar hyperplasia” in Chinese) and “增生性瘢痕” (“hypertrophic scars” in Chinese) to search on the Chinese version of TikTok (“Douyin”) on February 28, 2024, with the default sorting option of “overall ranking.” To avoid bias caused by personalized recommendations, we used newly registered accounts to conduct searches. We did not apply any filtering conditions to restrict the search. Consumers seeking general health videos typically do not scroll very far when searching online; they usually browse only the first few pages of search results. Furthermore, videos that rank low in the search results of the “overall ranking” mode often have little relevance to the topic [29]. Considering the aforementioned situation, we selected the top 100 videos for further analysis of the search results. Subsequently, we excluded non-Chinese, irrelevant, repetitive, and silent videos, resulting in 153 videos selected for the final data analysis (Figure 1).

Exclusion criteria were non-Chinese videos and repetitive videos—which refers to videos with the same content but different sources; we based our judgments on the video descriptions and main content. We excluded silent videos, which are defined as content that consists only of images or text, with no voice or background sound. We determined whether a video was silent by overseeing each clip to ensure no audio. Moreover, we excluded irrelevant videos, which refers to videos that do not pertain to the themes of “scar hyperplasia” or “hypertrophic scars.” Examples included advertisements, entertainment videos, or content related to other health topics. We based our judgments on the video descriptions and main content.

**Figure 2:**
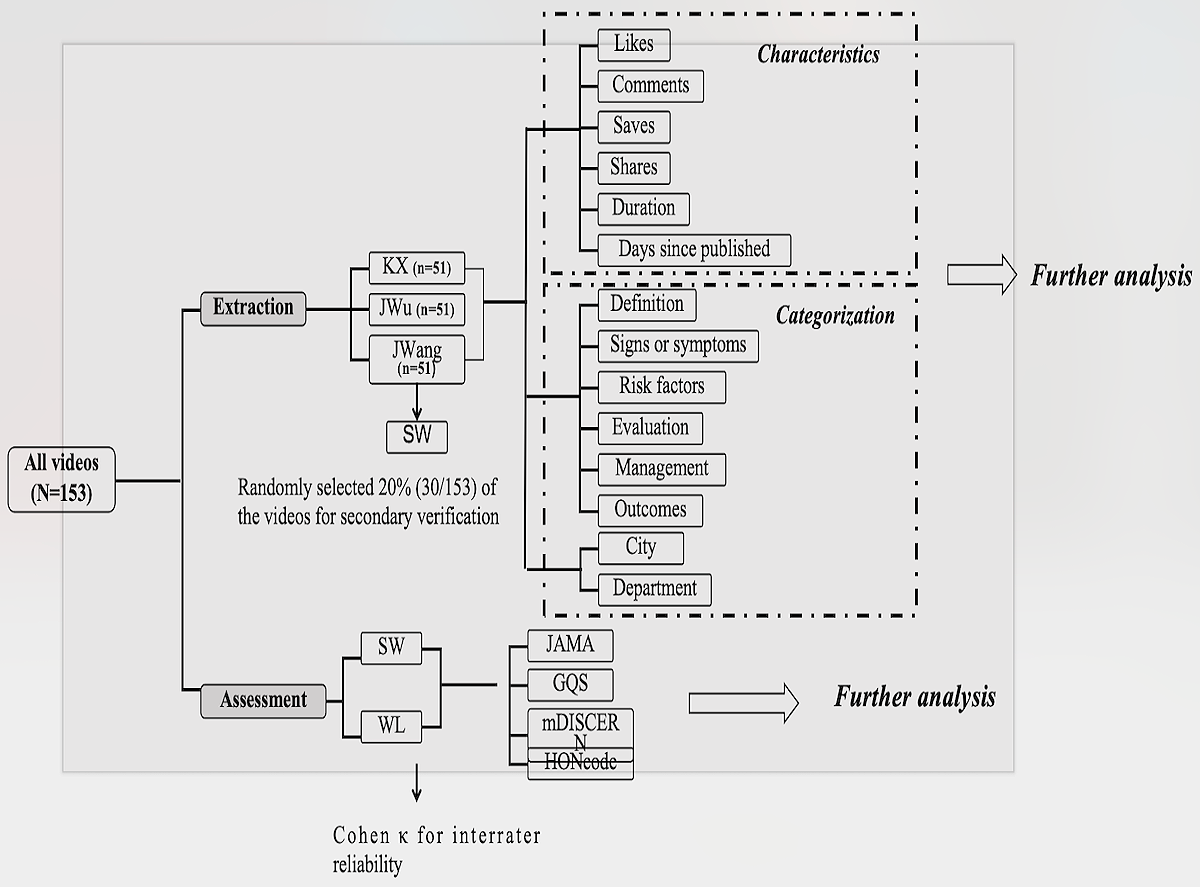
Flowchart for data extraction and analysis. GQS: Global Quality Scale; HONcode: Health on the Net Foundation Code of Conduct; *JAMA*: *Journal of the American Medical Association*; mDISCERN: modified DISCERN.

We extracted data directly from the public information provided by the TikTok platform, as it lacks a bulk data export function. Consequently, we manually recorded the relevant data for each video. Team members used browser tools, including screenshots and text-copying functions, to transfer video information into Microsoft Excel spreadsheets for further classification and analysis. Three team members (J Wu, KX, and J Wang) completed the data extraction, each responsible for a specific portion of the videos. To ensure the accuracy of the data entry, we developed a unified operations manual, and cross-checking was conducted by another team member (SW) after the data entry was finished. In addition, we randomly selected 20% (30/153) of the videos for secondary verification, which resulted in a data consistency rate of over 95% (Figure 2).

### Video Classification

**Figure 3:**
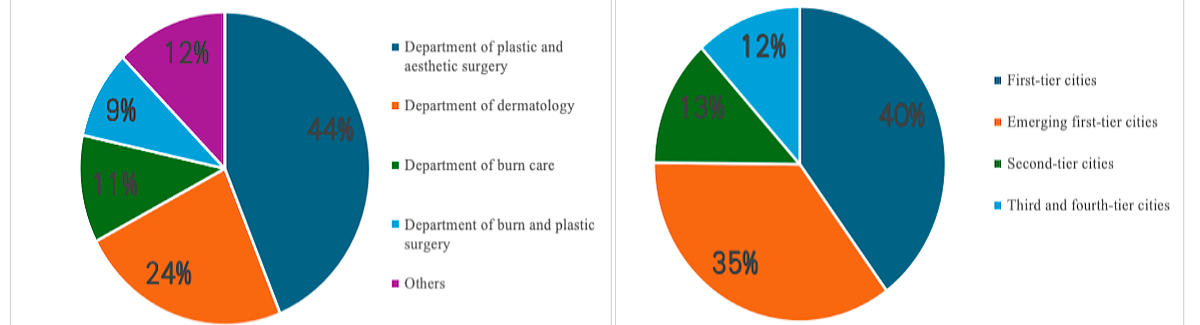
Percentage of videos on health care professionals from different departments and city tiers.

The content of the videos was classified through manual review. Three authors (J Wu, KX, and J Wang) independently watched each video and categorized it into 1 of the following 6 groups based on its content: the definition, signs and symptoms, risk factors, evaluation, management, or outcomes. All videos were provided by health care professionals; we further classified them according to department categories, specifically including plastic and aesthetic surgery, dermatology, burn care, burn and plastic surgery, and a general category termed “other departments,” which includes various departments, such as traditional Chinese medicine and pediatric surgery. In addition, to comprehensively consider the distribution of video resources, we categorized the videos based on city administrative levels, including first-tier cities, emerging first-tier cities, second-tier cities, third-tier cities, and fourth-tier cities, to reflect regional differences more accurately (Figures 2 and 3, Tables 1 and 2) [30].

**Table 1:** Scoring criteria and weight for categorization of cities.

Scoring criteria			Weight
**Commercial resource concentration**			0.19
	Big Brand Favorability Index	0.32	
	Commercial Core Index	0.40	
	Commercial support maturity	0.28	
**Urban hub connectivity**			0.2
	Transportation connectivity	0.33	
	Talent Mobility Index	0.27	
	Industry Synergy Index	0.20	
	Commercial resource regional centrality	0.2	
**Urban population activity**			0.22
	Consumption activity	0.38	
	Social activity	0.31	
	Nighttime activity	0.31	
**New economy competitiveness**			0.20
	Corporate leadership	0.36	
	New Consumption Index	0.32	
	Industry Chain Ecosystem Index	0.32	
**Future flexibility**			0.19
	Innovation Atmosphere Index	0.32	
	Talent Attraction Index	0.39	
	City Size Index	0.29	

**Table 2:** The 2024 China city classification rankings.

City classification	List of cities
First-tier cities	Shanghai, Beijing, Shenzhen, and Guangzhou
Emerging first-tier cities	Chengdu, Hangzhou, Chongqing, Suzhou, Wuhan, Xi’an, Nanjing, Changsha, Tianjin, Zhengzhou, Dongguan, Wuxi, Ningbo, Qingdao, and Hefei
Second-tier cities	Foshan, Shenyang, Kunming, Jinan, Xiamen, Fuzhou, Wenzhou, Changzhou, Dalian, Shijiazhuang, Nanning, Harbin, Jinhua, Nanchang, Changchun, Nantong, Quanzhou, Guiyang, Jiaxing, Taiyuan, etc.
Third-tier cities	Urumqi, Linyi, Haikou, Huzhou, Yangzhou, Yancheng, Luoyang, Tangshan, Jining, Langfang, Taizhou, Ganzhou, Hohhot, Zhenjiang, Wuhu, Shantou, Handan, Jiangmen, Zibo, Yinchuan, etc.
Fourth-tier cities	Zhoushan, Qingyuan, Quzhou, Zhumadian, Deyang, Yibin, Longyan, Rizhao, Hongzhi, Anshan, Maoming, Binzhou, Qinhuangdao, Jilin, Kaifeng, etc.

The weights of the primary and secondary dimensions of the ranking were determined through scoring by the expert committee of the New First-Tier Cities Research Institute, while the indicators mentioned after the secondary dimensions were calculated using the principal component analysis method. The indicators for each subdimension of the ranking were primarily derived from data collected throughout 2023 or up to early 2024.

In this study, to determine the geographic location of video creators, we mainly obtained relevant information through 2 channels. First channel was user profile information, where the TikTok account profile of the video uploader usually voluntarily disclosed location information, such as city name or workplace. We manually checked the account home page of each uploader and recorded the geographic location mentioned in their profile (such as a hospital in Beijing). The second channel was the certification identification and employer information, where the TikTok platform usually included the publisher’s employer and department information for certified accounts. For example, the authentication information may have included “burn and plastic surgery department of a hospital in Shanghai” or “dermatology department of a hospital in Guangzhou,” based on which we could determine the geographic location of the uploader.

### Assessment of Video Content, Quality, and Reliability

We used the 6 questions developed by Goobie et al [31] to assess video content, focusing on disease definition, signs and symptoms, risk factors, evaluation, management, and outcomes. The hexagonal radar chart is a unique statistical tool that can display data from 6 different fields at the same time. Each dataset is mapped onto a separate axis, and the data points on each axis are connected by continuous lines to form a hexagonal outline. The main goal of this chart design is to visually represent the focus and impact weight of a specific subject, such as video content, across 6 core dimensions [19]. By doing so, the hexagonal radar chart simplifies the comprehension of complex data and provides a clear and user-friendly visual representation for both users and researchers [12,32].

The videos’ reliability and quality were assessed using 4 standardized evaluation tools: modified DISCERN (mDISCERN; Table 3), Global Quality Scale (GQS; Table 4), the *Journal of the American Medical Association* (*JAMA*; Table 5), and the Health on the Net Foundation Code of Conduct (HONcode; Table 6). We used a multifaceted approach to assess the quality and reliability of the educational content of the videos collected, mainly based on the following considerations:

- Each benchmark focuses on different dimensions. mDISCERN assesses the information quality, GQS evaluates overall content quality, *JAMA* evaluates the reliability of the video, and the HONcode examines the ethics and credibility of health information.
- By integrating multiple benchmarks, it is possible to more comprehensively capture the differences in quality and reliability dimensions in short videos.
- The multibenchmark method improves the objectivity of evaluation and avoids bias that may arise from a single benchmark.

**Table 3:** Description of modified DISCERN (mDISCERN) for evaluating the quality of the videos with information on hypertrophic scars.

mDISCERN	Scores (1 point is given for every yes and 0 points for no)
Is the video clear, concise, and understandable?	0-1
Are reliable sources of information used? (ie, publication cited and speaker is a specialist)	0-1
Is the information presented balanced and unbiased?	0-1
Are additional sources of information listed for patient reference?	0-1
Are areas of uncertainty or controversy mentioned?	0-1

**Table 4:** Description of the Global Quality Scale (GQS) for evaluating the quality of the videos with hypertrophic scars information.

GQS	Scores (range from 1=poor quality to 5=excellent flow and quality)
The information is of poor quality, and the flow of the site is poor. Most information is missing and not useful for patients at all.	1
The information is generally of poor quality and flow. Some information is listed, but many important topics are missing, and it is of very limited use to patients.	2
Moderate quality and suboptimal flow: Some vital information is adequately discussed, but other topics are poorly discussed and somewhat useful for patients.	3
Good quality and flow: Most relevant information is listed, but some topics still need to be covered. It is useful for patients.	4
The information is of excellent quality and has excellent flow. It is beneficial for patients.	5

**Table 5:** Description of the *Journal of the American Medical Association (JAMA)* for evaluating the quality of the videos with hypertrophic scar information.

*JAMA* benchmark criteria	1 point for each criterion, with a total score of 4 points
Authorship	Author and contributor credentials and their affiliations should be provided.
Attribution	All copyright information should be clearly listed, and references and sources for content should be stated.
Currency	The initial date of posted content and subsequent updates to the content should be provided.
Disclosure	Conflicts of interest, funding, sponsorship, advertising, support, and video ownership should be fully disclosed.

**Table 6:** Description of the Health on the Net Foundation Code of Conduct (HONcode) for evaluating the quality of videos with information about hypertrophic scars.

HONcode	Detail
Authority	Any medical or health advice given in the video must come from a qualified health professional unless it is clearly stated that the information does not come from a qualified health source.
Complementarity	The information provided in the video must be designed to support the patient’s HTS^a^ self-management, but it is not meant to replace the patient-physician relationship.
Privacy policy	The information in the video maintains the right to confidentiality and respect of the individual patient featured.
Referenced and dated	Each video contains references to source data on information presented or contains a specific HTML link to source information.
Justifiability	Each video containing claims on the benefits or performance of specific skills and behaviors, interventions, treatments, products, etc must be supported by evidence through references or HTML links.
Transparency	The video must provide the viewer with contact information or a URL to more information.
Financial disclosure	Any individual or organization that contributes funds, services, or material in the posted video must be clearly identified in the video or video description.
Advertising policy	If an advertisement supports funding to the video or the video’s developers, it must be clearly stated. Included advertising must be clearly differentiable to the viewer. There should be a clear difference between the advertising material and the educational material in the video.

^a^HTS: hypertrophic scar.

mDISCERN is the most commonly used quality research tool [33]. This method has been widely used to evaluate information quality on video-sharing platforms [34]. Considering that the video studied belongs to the medical category, mDISCERN is based on the following 5 aspects: clarity, relevance, traceability, robustness, and fairness. The mDISCERN has 5 questions that need answers as “yes” or “no.” A score of 1 indicates yes, 0 indicates no, and the maximum score is 5 [35].

GQS was used to assess the overall content quality of the videos in this study. The GQS is a commonly used 5-point scale comprising 5 criteria ranging from 1 to 5, with higher scores indicating better quality [36-38].

*JAMA* was used to evaluate the reliability of the video [39]. The rating is according to the 4 predetermined issues: authorship, attribution, currency, and disclosure. There is 1 point for each criterion, with a total score of 4 points [40].

The HONcode consists of 8 issues that are predetermined for the rating: authority, complementarity, privacy policy, reference and date, justifiability, transparency, financial disclosure, and advertising policy [41,42]. The details of the scoring criteria are mentioned subsequently. First, any medical or health advice given in the video must come from a qualified health professional unless it is clearly stated that the information does not come from a qualified health source. Second, the information provided in the video must be designed to support the patient’s HTS self-management, but it is not meant to replace the patient-physician relationship. Third, the information in the video maintains the right to confidentiality and respect of the individual patient featured. Fourth, each video contains references to source data on the information presented or contains a specific HTML link to source information. Fifth, each video containing claims on the benefits or performance of specific skills or behaviors, interventions, treatments, products, etc must be supported by evidence through references or HTML links. Sixth, the video must provide the viewer with contact information or a URL to more information. Seventh, any individual or organization that contributes funds, services, or material in the posted video must be clearly identified in the video or video description. Eighth, if an advertisement supports funding to the video or the video’s developers, it must be clearly stated. Included advertising must be differentiable to the viewer: There should be a clear difference between the advertising material and the educational material in the video. There is 1 point for each criterion, with a total score of 8 points.

Although *JAMA* and HONcode are commonly used to evaluate formal or long-format medical content (such as websites, journals, or organizational publications), their application has been extended to user-generated content on the TikTok short video platform [43]. In this study, we had a detailed discussion on the benchmark before scoring and adjusted the scope of application of the scoring criteria. For the “disclosure” rating item, we focused on whether the video identified the publisher’s identity and affiliation rather than detailing funding sources or advertising disclosures. Regarding the “citation source” standard, many videos did not provide explicit references and often used vague terms, such as “research shows” or “experts say.” To tackle this issue, we reached the following consensus: (1) videos that do not provide any source explanation will receive a score of 0; (2) content that mentions vague references (like “research shows”) but fails to specify the source will receive a score of 0.5, indicating a partially satisfied score; and (3) videos that list their sources or include relevant reference information in the video description will receive a score of 1. We also focused on evaluating whether the core medical information of the video was accurately conveyed based on the video duration limit rather than comprehensive coverage. In addition, during the rating process, we considered the background information of the video creator (such as certification marks or institutional affiliations) to help evaluate the credibility of the references.

The videos were evaluated by 2 qualified physicians (SW and WL) who have extensive experience in scar treatment. Before scoring the videos, the 2 evaluators reviewed the mDISCERN, GQS, *JAMA*, and HONcode scoring guidelines and conducted detailed discussions to prevent cognitive bias. The final score for each video was calculated by averaging the scores given by the 2 evaluators. If there was a significant difference between the scores of the 2 experts, the final score was determined through discussion with the third arbitrator (KX; Figure 2). In the evaluation process of 153 videos, Cohen κ values rated by experts showed high consistency (κ>0.80). Therefore, no case involved the third arbitrator.

### Ethical Considerations

All information used in this study came from publicly published TikTok (Douyin in Chinese) videos. This study did not involve clinical data, human specimens, or animal experiments, nor did it involve personal privacy. No personal data identifying the uploader’s identity, such as username or profile picture, were recorded or stored during the research process. Data analysis only focused on video content and interaction metrics (such as likes, comments, and shares). The study strictly abided by the terms of use of the TikTok platform and did not obtain the platform’s undisclosed data through any technical means. The research content did not involve any potential harm to user interests or platform rules and was only used for academic purposes. Therefore, this study did not require an ethics review.

### Statistical Analyses

The data were analyzed using SPSS Statistics (version 29; IBM Corp). Continuous variables were presented as medians with IQRs, while categorical variables were presented in terms of numbers and percentages. Cohen κ was used to measure interrater reliability between the 2 evaluators. According to the criteria set by Landis and Koch [44], a κ value > 0.8 indicates almost perfect agreement, a value between 0.6 and 0.8 indicates substantial agreement, a value between 0.4 and 0.6 indicates moderate agreement, and a value <0.4 indicates poor agreement. Spearman correlation analysis was conducted to assess the relationships between quantitative variables. A significance level of *P*<.001 was considered statistically significant.

## Results

### Video Characteristics

A total of 153 videos about scar hyperplasia and HTSs were found on Chinese TikTok, all posted by health care professionals. In terms of departmental distribution of video uploads, professionals from the department of plastic and aesthetic surgery contributed the highest proportion of video content, accounting for 67 videos (43.8%), followed by dermatology (n=36, 23.5%), burn care (n=17, 11.1%), burn and reconstructive surgery (n=14, 9.2%), and “other departments,” which included traditional Chinese medicine and pediatric surgery (n=19, 12.4%). Further analysis by city tier revealed significant differences in video publication volume. Health care professionals in first-tier cities were the most active, accounting for 61 (39.9%) of the video uploads, followed by new first-tier cities (n=54, 35.3%), second-tier cities (n=20, 13.1%), and third- and fourth-tier cities (n=18, 11.8%; Figure 3). The general characteristics of the videos are presented in Tables 7-9.

The median time since upload was 212 (IQR 54-321) days, and the average video duration was 43 (IQR 33-58, SD 36) seconds. All videos received a maximum of 21,000 likes (median 72, IQR 31-189), 1230 comments (median 9, IQR 4-32), 7580 favorites (median 21, IQR 7-66), and 2292 shares (median 20, IQR 9-63). Table 8 describes the critical features of the videos uploaded by health care professionals from different departments. Notably, videos posted by dermatologists stood out in several engagement metrics, specifically with higher numbers of likes (median 112.5, IQR 44.5-254), comments (median 12, IQR 5.75-46.25), saves (median 36.5, IQR 13.75-94.75), and shares (median 29, IQR 11.75-70.75). This phenomenon may reflect the public’s interest and preference for educational dermatology videos. Further analysis (Table 9), which focused on the essential characteristics of videos uploaded by health care professionals from different city tiers, revealed a notable phenomenon. Although some cities may not have the overall resource advantage, emerging first-tier cities’ videos showed unique appeal in user engagement. The median numbers of likes, comments, saves, and shares were 94 (IQR 37.75-183), 12 (IQR 5-55.5), 24.5 (IQR 8-78.75), and 26.5 (IQR 10.25-66), respectively. This finding suggested that video content dissemination strategies should focus more on regional characteristics and alignment with user needs. In addition, the shortest video was 13 seconds long, the longest was 282 seconds long, and the first video was uploaded 1047 days before our search. In contrast, the most recent video was uploaded the day before data collection.

**Table 7:** Characteristics of hypertrophic scar videos (N=153).

Parameters		Values
**Video source, n (%)**		
	Health care professionals	153 (100)
**Department classification, n (%)**		
	Department of plastic and aesthetic surgery	67 (43.8)
	Department of dermatology	36 (23.5)
	Department of burn care	17 (11.1)
	Department of burn and plastic surgery	14 (9.2)
	Other departments	19 (12.4)
**City classification, n (%)**		
	First-tier cities	61 (39.9)
	Emerging first-tier cities	54 (35.3)
	Second-tier cities	20 (13.1)
	Third- and fourth-tier cities	18 (11.8)
Likes, median (IQR)		22 (31-189)
Comments, median (IQR)		9 (4-32)
Saves, median (IQR)		21 (7-6)
Shares, median (IQR)		20 (9-3)
Duration (s), median (IQR)		44 (33-8)
Days since published, median (IQR)		159 (54-21)
*JAMA*^a^ score, median (IQR)		1 (1-2)
GQS^b^ score, median (IQR)		2 (2-3)
mDISCERN^c^ score, median (IQR)		2 (2-3)
HONcode^d^ score, median (IQR)		3 (3-4)

^a^*JAMA*: *Journal of the American Medical Association*.

^b^GQS: Global Quality Scale.

^c^mDISCERN: modified DISCERN.

^d^HONcode: Health on the Net Foundation Code of Conduct.

**Table 8:** Characteristics of hypertrophic scars in videos across different departments.

Variable	Department of plastic and aesthetic surgery (n=67), median (IQR)	Department of dermatology (n=36), median (IQR)	Department of burn care (n=17), median (IQR)	Department of burn and plastic surgery (n=14), median (IQR)	Others (n=19), median (IQR)	Overall (n=153), median (IQR)
Likes	52 (30.5-61.5)	112.5 (44.5-254)	70 (34-189)	62 (37.25-146.25)	96 (18.5-234)	22 (31-189)
Comments	8 (4-19)	12 (5.75-46.25)	7 (2-33)	8.5 (3-27)	10 (3.5-40)	9 (4-32)
Saves	17 (8-56.5)	36.5 (13.75-94.75)	22 (6-50)	14.5 (7-35.25)	45 (5-97.5)	21 (7-66)
Shares	14 (8-55.5)	29 (11.75-70.75)	22 (6-50)	16.5 (7.75-47)	32 (8.5-96.5)	20 (9-63)
Duration (s)	45 (33.5-59)	41.5 (30-55.25)	45 (5-97.5)	43.5 (29.5-55.75)	53 (42.5-58.5)	44 (33-58)
Days since published	16.5 (39.5-334)	143.5 (71.5-281)	162 (88-196)	258 (69.25-464)	145 (28-260)	159 (54-321)
*JAMA*^a^ score	1 (1-1.5)	1.75 (1-2)	1.5 (1-2)	1 (1-1)	1 (1-1.5)	1 (1-2)
GQS^b^ score	2 (2-3)	2 (2-3)	2 (2-2.5)	2 (2-2.75)	2 (2-3)	2 (2-3)
mDISCERN^c^ score	2 (2-3)	2 (2-3)	3 (2-3)	2 (2-3)	2 (2-2)	2 (2-3)
HONcode^d^ score	3 (3-4)	3 (3-3)	3 (3-4)	3.25 (3-4)	3 (3-3.5)	3 (3-3.75)

^a^*JAMA*: *Journal of the American Medical Association*.

^b^GQS: Global Quality Scale.

^c^mDISCERN: modified DISCERN.

^d^HONcode: Health on the Net Foundation Code of Conduct.

**Table 9:** Characteristics of hypertrophic scars in videos across different city tiers.

Variable	First-tier cities (n=61), median (IQR)	Emerging first-tier cities (n=54), median (IQR)	Second-tier cities (n=20), median (IQR)	Third- and fourth-tier cities (n=18), median (IQR)	Overall (n=153), median (IQR)
Likes	79 (34-220)	94 (37.75-183)	53.5 (29.5-108.75)	51.5 (24-144.25)	22 (31-189)
Comments	8 (4-33)	12 (5-45.5)	10.5 (3-15)	10.5 (4-16.5)	9 (4-32)
Saves	22 (8-76)	24.5 (8-78.75)	9 (4-52.5)	13 (8-46)	21 (7-66)
Shares	20 (9-63)	26.5 (10.25-66)	18 (7.75-47.25)	10 (7-40)	20 (9-63)
Duration (s)	41 (31-55)	43 (30.25-54.5)	50 (38.25-60.5)	58 (37-102)	44 (33-58)
Days since published	188 (80-317)	119.5 (54.75-325.5)	156.5 (76.75-329)	25 (14-168)	159 (54-321)
*JAMA*^a^ score	1 (1-2)	1 (1-2)	1 (1-1)	1.25 (1-2)	1 (1-2)
GQS^b^ score	2 (2-3)	2 (2-3)	2 (2-2)	2 (2-3)	2 (2-3)
mDISCERN^c^ score	2 (2-3)	2 (1-3)	2 (2-3)	2.5 (2-3)	2 (2-3)
HONcode^d^ score	3 (3-4)	3 (3-4)	3 (3-4)	3 (3-4)	3 (3-4)

^a^*JAMA*: *Journal of the American Medical Association*.

^b^GQS: Global Quality Scale.

^c^mDISCERN: modified DISCERN.

^d^HONcode: Health on the Net Foundation Code of Conduct.

### Analysis of Video Content

**Figure 4:**
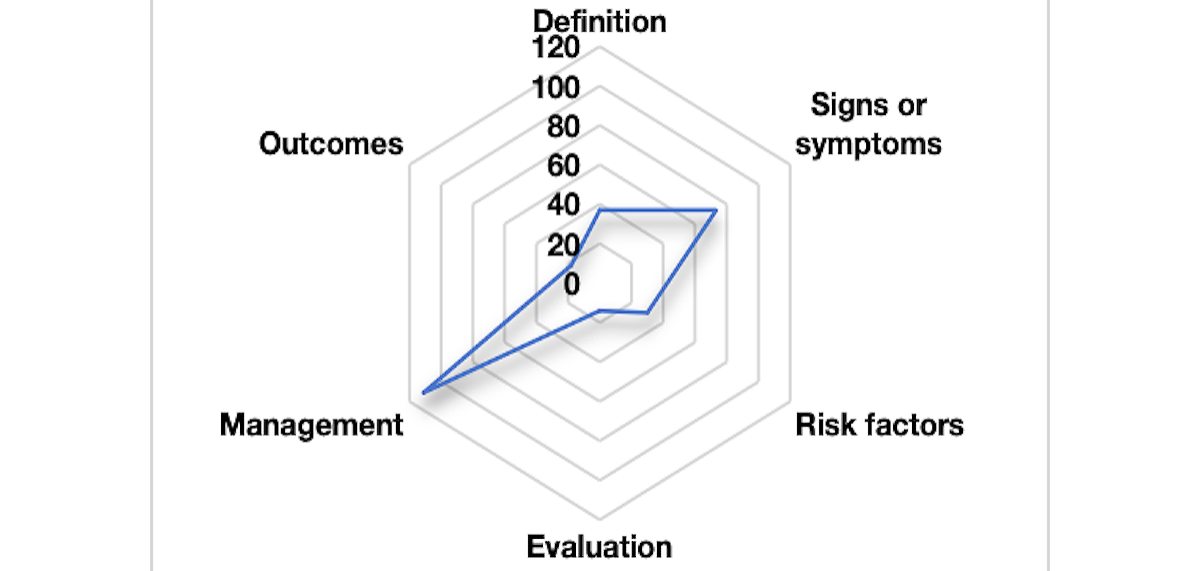
Hexagonal radar charts of the content of videos on hypertrophic scars.

**Figure 5:**
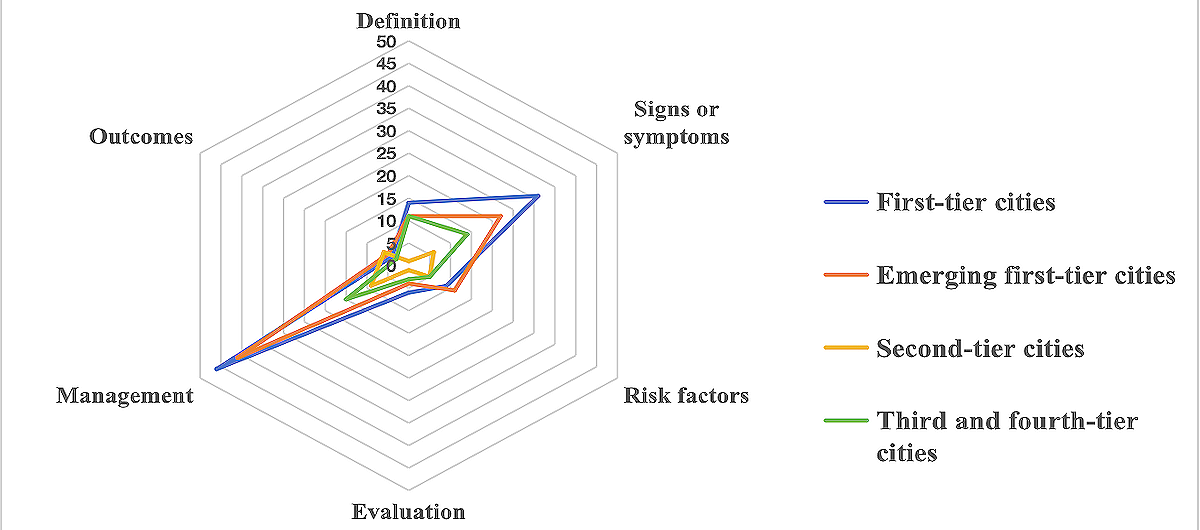
Hexagonal radar charts of the content of different city-tier videos on hypertrophic scars.

According to the hexagonal radar chart, the most frequently discussed topic in all videos was managing HTSs, which appeared in approximately 72.5% (111/153) of the videos. This was followed by the symptoms and definitions of HTSs mentioned in 47.7% (73/153) and 24.2% (37/153) of the videos. However, the outcomes and risk factors of HTSs should have been addressed, with only 11.8% (18/153) and 19.6% (30/153) of the videos discussing these aspects. The least mentioned topic was the evaluation of HTSs, with only 9.2% (14/153) of the videos adequately covering evaluation, while 90.8% (139/153) of the videos provided little to no information on this aspect (Figure 4). An in-depth analysis of the hexagonal radar chart structures presented by various city tiers revealed a common phenomenon—regardless of city tier, the video content predominantly focused on managing HTSs, while the evaluation of HTSs was notably less addressed (Figure 5). This finding aligned with our overall evaluation of the video content, further confirming the distribution bias of video resources toward specific topics.

### Assessment of Video Quality

We found that the median *JAMA* score for all uploaded TikTok videos was 1 (IQR 1-2). When we used the mDISCERN score to assess the usability and reliability of the videos, the median score was 2 (IQR 2-3). Specifically, the median GQS score for the overall quality of the TikTok videos was 2 (IQR 2-3), while the median HONcode score was 3 (IQR 3-4).

To explore whether health care professionals from different departments and cities influenced the quality and reliability of videos, we conducted a detailed categorization based on departmental affiliations and city tiers. The results showed slight differences in the quality scores, specifically *JAMA*, GQS, mDISCERN, and HONcode, among videos uploaded from 1 to 3(IQR 1.75-2.25), indicating an overall low quality of the videos. There was little variation in video quality ratings among different city levels; HONcode ratings were primarily concentrated at 3 (IQR 3-4) points, suggesting moderate overall content quality. Further analysis revealed that videos related to burn plastic surgery and burn surgery had relatively high-quality ratings, with HONcode score of 3.25 and an mDISCERN rating of 3. These departments exhibited stronger professionalism and scientific content. However, the videos suffered from insufficient interactivity, indicated by fewer likes and shares, resulting in a lower dissemination effect compared to dermatology videos.

For new first-tier cities, the video quality ratings (GQS: 2 and mDISCERN: 2) were comparable to those in first-tier cities. In addition, the median upload time for these videos was shorter (119.5 days compared to 188 days), indicating that the content was timelier. In contrast, videos from third- and fourth-tier cities achieved HONcode ratings of 3 and mDISCERN ratings of 2, showing no significant disadvantage in terms of quality. However, these cities had lower upload volumes and interaction metrics, averaging 51.5 likes and 10.5 comments. This could be attributed to limited medical resources and a smaller number of creators in those areas.

### Correlation Analysis

The nonnormal distribution of the data led us to use Pearson correlation analysis to investigate the relationships between different video variables and all evaluation scores (Table 10). We found that each video variable positively correlated with the scores obtained from the 4 evaluation methods. Notably, likes, comments, favorites, and shares were the only variables that showed significant correlations with all evaluation scores (*P*<.001), indicating that higher-quality videos tended to be more appreciated by viewers. Specifically, the number of days since video upload was significantly positively correlated only with GQS scores (*r*=0.393; *P*<.001) and mDISCERN scores (r=0.273; *P*<.001). In contrast, video duration did not significantly correlate with the evaluation scores (Table 10).

**Table 10:** Pearson correlation analysis between the video variables and all evaluation scores.

Variables			*JAMA* ^a^		GQS^b^		mDISCERN^c^		HONcode^d^
**Likes**									
	*r*	0.514^e^		0.740^e^		0.394^e^		0.287^e^	
	*P* value	<.001		<.001		<.001		<.001	
**Comments**									
	*r*	0.403^e^		0.613^e^		0.438^e^		0.426^e^	
	*P* value	<.001		<.001		<.001		<.001	
**Saves**									
	*r*	0.504^e^		0.736^e^		0.424^e^		0.293^e^	
	*P* value	<.001		<.001		<.001		<.001	
**Shares**									
	*r*	0.470^e^		0.701^e^		0.413^e^		0.301^e^	
	*P* value	<.001		<.001		<.001		<.001	
**Days since published**									
	*r*	0.123		0.393^e^		0.273^e^		0.098	
	*P* value	.11		<.001		<.001		.23	
**Duration**									
	*r*	0.023		0.105		0.149		0.169	
	*P* value	.78		.20		.07		.04	

^a^*JAMA*: *Journal of the American Medical Association*.

^b^GQS: Global Quality Scale.

^c^mDISCERN: modified DISCERN.

^d^HONcode: Health on the Net Foundation Code of Conduct.

^e^The correlation is significant at a significance level of .01 (2-tailed).

In addition, we used Spearman correlation analysis to reveal the relationships between different video variables. We observed a positive correlation between the following variables: likes and comments (ρ=0.777; *P*<.001), likes and saves (ρ=0.941; *P*<.001), likes and shares (ρ=0.904; *P*<.001), likes and uploads (ρ=0.534; *P*<.001), comments and saves (ρ=0.781; *P*<.001), comments and shares (ρ=0.820; *P*<.001), comments and uploads (ρ=0.404; *P*<.001), saves and shares (ρ=0.897; *P*<.001), saves and uploads (ρ=0.499; *P*<.001), and shares and uploads (ρ=0.564; *P*<.001). Meanwhile, there was no significant relationship between video duration and other variables (Table 11).

**Table 11:** Spearman correlation analysis between the video variable.

Variable		Likes	Comments	Saves	Shares	Days since published	Duration
**Likes**							
	ρ	1	0.777^a^	0.941^a^	0.904^a^	0.534^a^	0.072
	*P* value	—^b^	<.001	<.001	<.001	<.001	.38
**Comments**							
	ρ	0.777^a^	1	0.781^a^	0.820 ^a^	0.404 ^a^	0.122
	*P* value	<.001	—	<.001	<.001	<.001	.13
**Saves**							
	ρ	0.941^a^	0.781^a^	1	0.897^a^	0.499^a^	0.105
	*P* value	<.001	<.001	—	<.001	<.001	.20
**Shares**							
	ρ	0.904^a^	0.820^a^	0.897^a^	1	0.564^a^	0.072
	*P* value	<.001	<.001	<.001	—	<.001	.38
**Days since published**							
	ρ	0.534^a^	0.404^a^	0.499^a^	0.564^a^	1	0.020
	*P* value	<.001	<.001	<.001	<.001	—	.80
**Duration**							
	ρ	0.072	0.122	0.105	0.072	0.020	1
	*P* value	.38	.13	.20	.38	.80	—

^a^The correlation is significant at a significance level of .01 (2-tailed).

^b^Not applicable.

## Discussion

### Principal Findings

Health problems are crucial and need daily attention, accurate assessment, and timely intervention. With the increasing popularity of the mobile internet, it has become one of the most popular ways to obtain health and medical information. A survey shows that 70% of internet users rely on the internet as their primary source of health information [45]. In this cross-sectional study, we used *JAMA* GQS, mDISCERN, and HONcode tools to evaluate the quality and reliability of HTS-related videos on the Chinese version of TikTok (Douyin). The results showed that the quality and reliability of HTS-related videos from TikTok were generally moderate. From the perspective of video sources, HTS-related videos were mainly released by health professionals. TikTok has strict verification rules to protect users’ interests, information security, and content reliability, and it requires only certified institutions or individuals to share medical-related videos on the platform [46]. In terms of video content, the video integrity was insufficient. Most (111/153, 72.5%) of the videos were related to HTS management. From the perspective of video classification, compared with other departments and cities, the videos uploaded by health professionals in burn departments and burn plastic-surgery departments, and videos produced in first-tier and emerging first-tier cities, were of slightly higher quality.

Users should exercise caution when seeking information on HTSs from TikTok. It is advisable to choose videos uploaded by health care professionals from burn departments and burn plastic surgery departments, and in the Chinese context, those produced in first-tier and emerging first-tier cities.

### Analysis of Overall Video Quality and Correlation

Our research uncovered an interesting phenomenon—only a few (1/153, 0.7%) videos thoroughly covered all aspects of HTSs, offering authoritative and practical guidance. Most (111/153, 72.5%) videos focused mainly on treatment methods, with symptom descriptions coming next and preventive measures mentioned less frequently. This may be related to the format of the TikTok platform, where video lengths vary; however, according to the latest statistics, the average length of popular videos is about 40 seconds [47]. This characteristic requires creators to present health information within a limited time frame, thereby affecting the depth and coverage of the video, and encourages users to create multiple videos on the same topic, each focusing on different aspects [48]. Our findings support this observation. Because a single video cannot cover all 6 core aspects of HTSs due to time constraints, users tend to split these into multiple videos presented as a series [46]. However, social media platforms usually recommend videos based on algorithms or randomness, making it difficult for users to access comprehensive health information systematically [49].

In the evaluation process of 153 videos, *JAMA*, GQS, mDISCERN, and HONcode scale values rated by experts showed high consistency (Cohen κ>0.80). Most videos on this platform did not receive high scores based on evaluations using *JAMA*, GQS, mDISCERN, and HONcode scales. This suggests that short videos about HTSs have poor quality and reliability. According to the recommendation algorithm of TikTok, people may primarily watch recently uploaded videos, and longer videos might cause viewers to lose patience and interest, leading to video skips. In addition, this mechanism determines that videos with more likes are more likely to be recommended; therefore, popular videos with lower quality have become more popular, further exacerbating the gap between video quality and popularity. We also found that videos from third- and fourth-tier cities received higher scores; however, this result only partially reflects the quality and reliability of their video content. The main reason is the relatively limited sample size from third- and fourth-tier cities, which may introduce some statistical bias. Therefore, caution is needed when interpreting these scores to avoid misinterpretation or misleading conclusions. To address this issue, we recommend that short video platforms introduce professional certification for experts and use unique markers to improve the trustworthiness of medical video content and reduce the spread of misinformation. The review standards for content uploaders on short video platforms are not yet comprehensive and strict. A significant number of nonprofessionals are still posting medical and scientific videos, which, to some extent, affects the accuracy and authority of the content [19]. Therefore, platforms should improve their verification and management procedures to ensure the professionalism and reliability of medical videos.

Our research discovered a potential link between video attributes and evaluation scores. We found a positive relationship between video length and evaluation scores, indicating that longer videos may improve quality by offering more informative content. However, this correlation was not statistically significant (*P*>.05). Previous studies have suggested that high-quality videos are often longer, which is consistent with our findings [50,51]. Excessively long videos might decrease viewer interest, resulting in fewer views, likes, and user engagement. This decrease in interest may stem from reduced viewer motivation despite the comprehensive content [52]. Therefore, publishers should consider video length carefully to maintain viewer interest and effectiveness of dissemination while upholding content quality. In addition, metrics, such as likes, comments, favorites, and shares, can gauge video popularity. Our analysis found significant positive correlations (*P*<.001) between these metrics and evaluation scores, indicating that high-quality videos are more likely to receive viewer approval. This finding aligns with the research conducted by Kong et al [12], which evaluated the quality of TikTok videos focused on diabetes health education. Their study found that higher-quality videos tend to receive greater recognition from audiences, evidenced by increased praise and sharing rates. In addition, our research revealed a positive correlation between the upload time of videos and their quality ratings, such as the GQS and mDISCERN scores (*P*<.001). This suggests that audiences prefer more timely and relevant content. Similar to the findings by Kong et al [12], we observed that the upload timing of videos is positively correlated with user engagement. However, in contrast to the work of Kong et al [12] and other studies that examined YouTube videos as sources of health information, our research found that TikTok videos received lower overall quality ratings. Specifically, in this study, the median *JAMA* score for TikTok videos was 1 (IQR 1-2), while YouTube videos typically received higher ratings in comparable studies (Kong et al [12] reported a median score of 2.5). This disparity may be attributed to the distinct characteristics of each platform. The short video format of TikTok, usually limited to 40 seconds, restricts the depth of content, whereas YouTube allows for longer videos that are more likely to adhere to *JAMA* and HONcode standards for comprehensive information. Furthermore, we found that interaction metrics for TikTok videos, such as likes and shares, were significantly correlated with GQS and mDISCERN ratings (*r*=0.740 and *r*=0.394, respectively; *P*<.001). This supports the conclusion made by Kong et al [12] that high-quality videos tend to engage audiences more actively. In addition, our study revealed variations in interaction metrics among medical professionals from different departments and cities, with videos uploaded from first-tier cities showing higher rates of likes and shares (*P*<.05). This finding has not been extensively explored in research on other platforms, suggesting that user behavior on TikTok may be influenced by unique regional and professional factors.

### Analysis of Evaluation Tools

This study comprehensively used *JAMA*, GQS, mDISCERN, and HONcode to evaluate the quality and reliability of TikTok short videos, mainly based on the considerations mentioned subsequently. First, each benchmark focuses on different dimensions. *JAMA* evaluates authorship and transparency, GQS evaluates overall content quality, mDISCERN focuses on information reliability, and the HONcode examines the ethics and credibility of health information. Second, by integrating multiple benchmarks, it is possible to more comprehensively capture the differences in quality and reliability dimensions in short videos. Third, the multibenchmark method improves the objectivity of evaluation and avoids bias that may arise from a single benchmark. However, we also recognize that these benchmarks were not originally designed for short videos and may pose applicability challenges. For example, *JAMA* and HONcode standards are commonly used to evaluate formal or long-format medical content (websites, journals, or organizational publications). However, this study attempts to extend their application to user-generated content on the TikTok short video platform. These videos mainly focus on visual effects and have a duration between 33 and 58 seconds, so they may not fully meet the requirements of *JAMA* standards for content depth and information transparency. To overcome this challenge, 2 scoring experts had a detailed discussion on the benchmark before scoring and adjusted the scope of application of the scoring criteria. For example, the “disclosure” rating item focuses on whether the video identifies the publisher’s identity and affiliation rather than detailing funding sources or advertising disclosures. The rating experts also focus on evaluating whether the core medical information of the video is accurately conveyed based on the video duration limit rather than comprehensive coverage. By adjusting the scope of application of these benchmarks (such as simplifying citation source standards), they still have reference value in evaluating the accuracy and credibility of core medical information in short videos. In addition, the review study by Li et al [53] indicates that mDISCERN is the most commonly used evaluation tool for health information videos. However, the review mainly focuses on long-format health education content and needs to explore the applicability of mDISCERN, specifically on short video platforms. Secondly, mDISCERN’s single benchmark may need to be able to cover the multidimensional quality assessment needs in short videos. Therefore, our study attempts to compensate for the dimensions that a single benchmark may overlook, such as video transparency and overall content quality, by combining other benchmarks, such as *JAMA* and GQS. Therefore, this study chooses to comprehensively use multiple benchmarks to evaluate the quality and reliability of short videos from different perspectives. Future research should further optimize and develop evaluation tools for short videos to enhance their applicability and scientific validity.

### Limitations and Future Directions

This research is the first to use 4 evaluation tools (*JAMA*, GQS, mDISCERN, and HONcode) to comprehensively evaluate the quality and reliability of high-frequency videos about HTSs on the TikTok platform. The study also includes an in-depth analysis of the relationship between video characteristics (likes, comments, favorites, and shares) and video quality. However, there are limitations to this study. First, the sample is limited to videos uploaded on the Chinese TikTok platform, which may limit the generalizability of the findings to other languages (such as English) and platforms (such as BiliBili). Despite focusing on Chinese TikTok, the research aligns with studies on videos from various platforms. Given the prevalence of HTS as a health issue, the findings may offer insights for video content in other languages and platforms (such as international versions of TikTok and YouTube). Second, there is a lack of standardized methods for evaluating health information video content on TikTok [46]. The study used 4 standardized evaluation tools due to their proven effectiveness in assessing video quality on media platforms and their previous use in studies evaluating TikTok video quality [54,55]. However, these assessments are somewhat subjective. Despite 2 raters confirming the scores and using Cohen κ to quantify interrater reliability, subjective differences cannot be ignored [20]. This highlights the need for the development of more suitable scoring standards. Third, limiting the analysis scope to verified accounts may result in certain limitations, such as not including videos published by ordinary users (such as patients) or unverified accounts. These videos may contain patients’ firsthand experiences or other nonprofessional information, which can impact the comprehensiveness of research conclusions. Future research should consider expanding the scope of analysis and adopting broader validation criteria to cover a more diverse range of video sources. Fourth, although we only selected videos uploaded by medical professionals certified by the platform, we cannot completely rule out the ambiguity of the author’s identity information. For example, some uploaders may not be the video’s actual creators or information providers but may only participate in video publishing. This uncertainty may affect the accuracy of *JAMA*’s benchmark “authorship” score, potentially leading to bias in research results. Fifth, there are inherent issues with viewing TikTok as a platform for disseminating health information. TikTok’s recommendation algorithm tends to push videos that easily attract attention rather than the most scientifically sound ones. This mechanism may lead to the dissemination of misleading or incomplete information. The subject of this study is limited to short videos related to HTSs on the TikTok platform, and all videos are uploaded by medical professionals. Although this choice ensures the scientific and credible nature of the video content, it also limits the generalizability of the research results. Videos related to HTSs uploaded by other groups, such as ordinary users or unverified health influencers, were not included in the analysis, which may limit the applicability of the research results to the broader dissemination of short videos related to health. In addition, the data source of this study is limited to Chinese TikTok (Douyin). The platform culture, user behavior, and regulatory policy may differ from the international version of TikTok or other social media platforms. Therefore, the research results may not directly apply to short video platforms in other countries or regions. In addition, TikTok’s video duration limit (usually within 40 s) poses a challenge to the comprehensiveness of health information. Although short videos on the platform can attract viewers to understand a topic quickly, their structure and depth often need to be improved, which may not meet the audience’s needs for complex health issues. Therefore, the effectiveness and limitations of TikTok as a tool for disseminating health information need to be further explored.

In addition, there are other evaluation criteria, such as the Video Popularity Index, that can be considered for assessing the quality of health-related information [56]. We recommend that future research incorporate various evaluation methods and platforms to assess the quality of video information more accurately.

The rapid advancement of internet technology and the rising health standards have led to the growing popularity of internet-based health promotion methods. Patients have shifted from being passive recipients to seeking health information actively [20]. With the widespread use of electronic devices, such as smartphones, and the flourishing multimedia technology, visual social media has become a crucial channel for accessing health information. However, the quality of video content varies greatly, leading to significant challenges. Some videos are misleading and provide inaccurate information to viewers, prompting professionals to advocate for stricter regulations. The Chinese government recently issued guidelines for media platforms to publish scientifically accurate health information, a move with global implications [57]. Enhancing the quality of health promotion videos has become a pressing issue requiring all stakeholders’ attention. A high-quality health promotion video should be scientifically accurate, appeal to a broad audience, and be easily understandable while eliminating any misleading content. Therefore, rigorous evaluation of video quality is essential to ensure the dissemination of reliable information. Future research should focus on constructing and optimizing platforms to better cater to the public’s health information needs.

### Conclusions

This research gathered 153 videos about HTSs from TikTok, a popular short video–sharing social media platform in China, and comprehensively evaluated their information quality. The findings revealed that the videos lacked reliable sources and content quality. Overall, videos on the topic of HTSs produced by health care professionals from the burn department and burn plastic surgery department as well as those from first-tier and emerging first-tier Chinese cities demonstrated more significant insights regarding quality and reliability. They provide audiences with more reliable medical information. Therefore, people may prefer content from these departments and cities when seeking information about HTSs. As video-sharing platforms become increasingly popular sources of health information, it is essential to improve regulation and quality control. Users should be cautious when seeking health care management information on short video platforms. To ensure access to accurate information on hypertrophic scarring, we recommend referring to professional and authoritative sources and platforms to safeguard health effectively.

## Data Availability

The datasets generated or analyzed during this study are available from the corresponding author on reasonable request.

## Abbreviations

GQS: Global Quality Scale
HONcode: Health on the Net Foundation Code of Conduct
HTS: hypertrophic scar
*JAMA*: *Journal of the American Medical Association*
mDISCERN: modified DISCERN
